# Report of Ocular Diseases, at the General Hospital, Fort Pitt, from 21st December, 1822, to 20th December, 1823

**Published:** 1824-10

**Authors:** George R. Melin

**Affiliations:** Assistant Surgeon to the Forces.


THE LONDON
### Medical and Physical Journal.

No. 4 of Vol. LII.] OCTOBER, 1824. [No. 308.

For many fortunate discoveries in medicine, and for the detection of numerous errors, the world is indebted to the rapid circulation of Monthly Journals; and there never existed any work, to which the Faculty, in Europe and America, were under deeper obligations, than to the Medical and Physical Journal of London, now forming a long, but an invaluable, series.—RUSH.

### ORIGINAL COMMUNICATIONS,
### SELECT OBSERVATIONS, &c.

ART. I.—*Report of Ocular Diseases, at the General Hospital, Fort Pitt, from 21st December, 1822, to 20th December, 1823.* By GEORGE R. MELIN, Assistant Surgeon to the Forces.

[Concluded from page 191.]

*Amaurosis.*—Of the nine cases admitted, there were none which deserve particular notice: they had all been long under treatment, and subjected to every plan that could be devised; so that, when they arrived here, their cases might be pronounced hopeless, as, in this complaint, I have seldom seen much relief afforded by any treatment, unless adopted in its commencement. Fortunately for the individuals who suffered in both eyes, the disease was only partial, and they enjoyed a useful degree of vision. Four of the cases were organic, and five functional; but, to this useful classification of Mr. Travers, the army practitioner has sometimes a third to consider,—namely, the feigned; and one which it is nearly as impossible to detect as to cure. The real disease is liable to degenerate into it; for the soldier who contracts a complaint which disqualifies him from further service, if he should be anxious to obtain his discharge, will be unwilling to acknowledge any amendment: and I know of no disease in which he can act thus, with so much impunity as in this complaint.

*Iritis.*—Only five cases of this complaint were admitted: three were discharged to their regiments, and two invalided. When the latter came into hospital, in one case the eye was disorganized, and in the other the retina was wholly insensible to light, and never afterwards recovered. In these cases, as well as in some that occurred after operation, I immediately commenced the use of mercury, and gave two grains of calomel, with a quarter of a grain of opium, every two hours, until the mouth became affected; on which a visible and marked improvement took place in the disease: the aqueous humour became clear, the absorption of effused lymph commenced, and the pupil recovered its round form.

Indeed, I know of no complaint in which the action of medicine is so clearly and beautifully seen as in this. I shall not enter into the question of the various species of iritis, whether idiopathic, syphilitic, or mercurial; but, in whatever form it appears, or whatever cause produces it, I must express my unqualified confidence in the power of mercury (when properly and judiciously administered,) to arrest its progress, and effect its cure. In general, I consider that, the more rapidly the system is placed under its influence, the better; and I have always commenced with two grains of calomel and a quarter of a grain of opium, in a pill, every two hours. But a case has lately occurred here, in a poor patient who applied for my advice, which will lead me to increase the quantity when next I meet with the complaint. The eye was painful, the sclerotic coat very vascular, the pupil irregular, the aqueous humour turbid, and a small quantity of lymph effused into the anterior chamber. I directed him to take the pills as above; and the next day he called on me, when, to my surprise, the eye was nearly perfectly recovered, and his mouth very sore. On inquiring I found that, by mistake, he had taken two pills every two hours. I ordered him to discontinue the pills, and directed a wash for the mouth; and on the following day there was no sign of the disease.

An interesting case of iritis occurred in an officer of the 40th, which Dr. Jones, Dr. Skey, and myself, considered to arise from a gouty habit. Mercury was quickly exhibited, and produced its usual beneficial influence on the iris; but an increased action in the sclerotic vessels, attended with pain and an increased flow of tears, taking place towards evening, and subsiding about five or six o'clock in the morning, regularly for a few days, led us to consider the complaint as having assumed an intermittent type. Bark was accordingly administered, and a pill of opium directed to be taken on the first appearance of the nocturnal paroxysm, and warm fomentations directed for the eye. Under this treatment, with strict attention to the state of the stomach and bowels, the nightly exacerbations were immediately relieved, and soon subsided. In about a month from the period of the first attack, the eye was perfectly restored to a healthy state.

Another case, which I think deserves to be noticed under this head, is one which occurred in one of the cases of cataract, but which I do not consider to have been caused by the operation, as the eye had perfectly recovered, and the inflammation did not appear until twenty-two days after it was performed.

The patient was of a delicate habit, and had not been long discharged from the medical division for a rheumatic attack, to which he was very subject. The inflammation of the iris came on during the evening, and, on visiting the hospital the following day, a small quantity of lymph was already effused into the anterior chamber. Calomel and opium were immediately directed, and in eight days the lymph was absorbed, and the eye recovered. A fortnight after, a similar attack took place, and by similar means in twelve days the eye was again recovered. Ten days after, the inflammation and deposition of lymph again occurred, and by the same means in nine days it was again overcome. Nine days after, the inflammation recurred, and in four days the report states the eye to have been recovered. Seven days afterwards, the same symptoms took place; but, as the effusion of lymph was but trifling, and all pain had subsided, and as the system was under the influence of mercury, it was not again given, and during the week the lymph became absorbed, and the iris resumed its natural appearance. On the seventh day after, a similar paroxysm of pain, attended by a flow of scalding tears, took place during the night, terminating before morning in an effusion of lymph in the bottom of the anterior chamber. The pain having entirely subsided, and the eye feeling perfectly easy, I determined to watch its progress, and to give only a small quantity of blue-pill at night, so as to keep up a slight mercurial action. In four days, the lymph was absorbed, and the iris became apparently healthy. On the seventh day, the attack again returned, with similar effects to the former ones. Considering it now, from the regularity of the periods and the great similarity of each attack, to be a species of intermittent, I ordered bark; and, on the day of the expected attack, the doses to be increased. On the seventh day, it returned; but the pain was less severe, and it did not occur at so early an hour as it generally did. During the week, the effused lymph became absorbed; but again, on the seventh day, the attack recurred. For three weeks it thus regularly returned, and the effusion of lymph became absorbed each week: but, finding that bark had no decided influence over it, I discontinued it, and directed six drops of liquor arsenicalis to be taken three times a-day, and on the day of the paroxysm to be increased to ten drops. This had the desired effect. The next attack was very mild: its duration was much diminished; and, in place of occurring in the beginning of the night, as formerly, it did not commence till morning. The following attack was still milder, and from that period he had no further return of them; and shortly afterwards he was discharged, in better general health than when admitted, and the eye quite recovered. He remained for some weeks at the depôt, before passing Chelsea Board, and the eye continued perfectly strong.

I have detailed this case thus fully, as it is the only one of the kind I ever met with; nor do I recollect to have ever read or heard of a similar one.

*Ulcers of Corneæ.*—These generally existed in the cases of chronic ophthalmia, and usually healed under the same treatment as adopted for that complaint. In some few cases it was necessary to touch them with a stronger solution of nitras argenti, and occasionally with the caustic itself; and in all cases of ulcers of the cornea I directed the eye to be covered with a bandage, so as to prevent the lids from disturbing the process of cicatrization by their motion. When ulcers of a spreading nature existed, I paid particular attention to the general state of health of the patient, as in most cases I found they depended on it. These species more frequently occurred in weak and delicate constitutions, or where the system had been much reduced by bleeding, purging, &c. In such cases, much benefit was derived, after clearing the primæ viæ, by bark and mineral acids, and gently touching the edges of the ulcers with a piece of caustic scraped to a fine point. When the ulcers were very irritable, I directed the eye to be held, every two or three hours, over the vapour of boiling water; to which was added some tincture of opium and camphor mixture, and which generally afforded much relief. By these means I succeeded in arresting the ulceration, and in not a single case did it extend through the cornea.

In the cases of rupture of the cornea and protrusion of iris admitted, if the protrusion was not large, I touched it, every second or third day, gently, with a pencil of caustic; but, if considerable, it was necessary to puncture the iris with a lancet, to allow the aqueous humour to escape, so as to reduce the tumor of the iris, before applying the caustic. This is generally sufficient to produce adhesion between the iris and cornea, and sometimes leaves very little deformity of pupil.

*Cataract.*—On the operations of the eye it will not be necessary to enlarge much: there was nothing very peculiar in the cases requiring them, nor in the mode of their performance; and it is the less requisite since the publication of the comprehensive and practical work on the subject by Mr. Guthrie, a work which comprises every thing that should be known by an operator, and which should be studied with attention by every surgeon who wishes to become a successful one.

A few of the operations for cataract and artificial pupil were performed by Mr. Schetky, with that adroitness and ability for which he is so distinguished, in every branch of his profession.

Of the eleven cases operated on, four were by extraction, and seven by cutting up the lens (for absorption) through the posterior chamber: the latter were cases in which both capsule and lens were opaque, and in which the operation by extraction was not admissible. In three of the unsuccessful cases, the operation itself was perfectly successful, but the retina proved to be insensible, which was strongly suspected in each case previous to the performance of the operation; but it was thought right to give the individual a chance for the recovery of vision.

In one of the cases it had the desired effect, in a very singular manner. The patient had amaurosis of the left eye and cataract, with suspected amaurosis of the right. The lens was extracted, without any benefit to the vision of the right eye; but, as soon as the eye was allowed to be exposed to the light, the retina of the left began gradually to recover its sensibility; and, when discharged from hospital, he enjoyed a very useful and comfortable degree of vision. The fourth unsuccessful case was one in which the vitreous humour was disorganized, and escaped as soon as the lens was extracted.

*Cataract and Contracted Pupil.*—In these three cases of contracted pupil and capsular cataract, the operation performed was that of making a central aperture in the iris, by a small knife passed through the sclerotic coat into the anterior chamber, and then cutting backwards through the iris, and breaking up the capsula and lens for absorption. Two of them succeeded remarkably well; but the third was followed by severe and repeated attacks of inflammation, which terminated in closure of the pupil, and thickening of the iris from effusion of lymph. The subject was an unfavourable one for operation; his constitution was worn out from tropical disease, and long service in the East Indies.

*Artificial Pupil.*—These operations were principally required in consequence of loss of vision, from cicatrix of the cornea opposite the pupil, or from rupture of the cornea and adhesion of theiris, with contracted pupil. Thirteen of them were performed by cutting out a portion of the iris, opposite a transparent part of the cornea; and one by the same operation as that described for cataract with artificial pupil.

In the operation by excision of the iris, care should be taken to unite the artificial with the natural pupil; for, if any of the circular fibres are left undivided, they will prevent the radiated ones from contracting, and the artificial pupil will be too small. This accident happened in one of my cases; and I was obliged to divide this band of fibres by a second operation, before a useful degree of vision was obtained.

None of the operations failed; but a few of them, as well as some for cataract, were followed by high inflammation, which was successfully subdued by general and local bleeding in the first instance, and then, if the iris appeared engaged, by calomel and opium every two hours, until the mouth became slightly affected. In the use of mercury in inflammation of the iris, succeeding operations, I place great reliance, and I am only surprised that it is not more generally adopted: I have found it of the greatest advantage, and am certain that I have saved many eyes by it, where bleeding alone would have failed.

*Entropion.*—Three of these operations were for inversions of the superior lids, and one was for that of the inferior. The one performed for the former was that described by Mr. Crampton, with the exception that the transverse incision on the inside the lid, uniting the two perpendicular ones, was carried through the tarsus, in place of barely dividing the conjunctiva. The case of entropion of the inferior lid was but slight, and only required the simple operation of removing an oval piece of the common integuments, and uniting the lips of the wound by sutures, to restore the lid to its natural position.

*Ectropion.*—These two cases appearing to me to possess some interest, both from their nature and the length of time they had existed, without any attempt being made to relieve them, I shall give in full, as they do not admit of being abridged. They clearly show that ectropion, dependent on cicatrization, can be cured by operation; and that we should attempt it in all such cases, whether caused by burns, scalds, ulceration, or wounds.

John Smith, 22d Foot, æt. 33; admitted 22d February, 1822, labouring under complete eversion of the lower lid of the left eye, occasioned by an extensive wound he received fourteen years ago, by the wheel of a cart passing over the side of his head, and fracturing the frontal bone and the external margin of the orbit. The edge of the everted tarsus adheres to the malar bone by a firm cicatrix, and the muscles of the cheek exercise great power in drawing it downwards, whenever the lower jaw moves. He has been in this state fourteen years, and suffers much inconvenience from it.

23d.—The operation for ectropion performed, by making a free horizontal incision beneath the whole ciliary margin of the lid, and thus detaching it from its firm adhesion to the malar bone; after which, a triangular piece was cut out of the centre of the tarsus, and the lips of the wound brought together by two sutures. The lid was then retained in its natural position by adhesive straps and a bandage.

25th.—The dressings removed this morning: the granulations of the horizontal wound healthy. Has experienced no pain since the operation.

27th.—The wound of the tarsus perfectly healed; the sutures removed. Granulations spring up rather too abundantly from the horizontal incision beneath the orbit, and were therefore touched with the sulphate of copper.

March 6th.—The wound nearly healed.

10th.—Discharged: the lid perfectly restored to its natural position.

Joseph Geoffry, Royal African Corps, æt. forty-seven; admitted 27th March, 1822, labouring under ectropion of the left inferior lid, occasioned by a sabre wound received ten years ago, which divided the orbital ridge of the superior maxillary and malar bones, and partially the nasal ones. The ciliary margin of the lid is held down by a firm cicatrix to the lower margin of the orbit, especially towards its inner angle, and he suffers a great deal of inconvenience from the dust and wind, as well as from the tears flowing over the lid. Conjunctiva not thickened.

The operation for ectropion was performed on the 9th of April, by detaching the everted margin of the lid from its adhesions, cutting out a triangular portion of the tarsus near its centre, and then bringing the divided edges together by means of two sutures. Great difficulty was encountered in dividing the adhesions, from their very firm nature, and the depth at which they existed within the margin of the orbit, and also from their being situated very near to the lachrymal sac, which required the dissection to be performed with great care, to avoid wounding it. This was, however, effected. The wound was then filled with lint, supported by adhesive straps, and the whole covered with simple dressing and a bandage.

12th.—The wound of the tarsus united, and the transverse one granulating freely. Has had no pain since the operation. The sutures were removed, and the wound filled with lint, and supported by straps.

16th.—The wound filling rapidly with healthy granulations, and the lid now retains its natural position.

20th.—The wound very nearly healed.

27th.—Discharged: the wound healed, and the eyelid perfectly retained in its natural situation.

Having now concluded the report of the practice pursued in the treatment of ophthalmic complaints at this hospital, I have the satisfaction to add, in support of its efficacy, that, out of 568 cases of the disease treated, there was not an eye lost, nor one impaired, that was not irrecoverably so before admission into hospital; and that, in thirty-seven cases of operation, only one can be fairly said to have failed. In conducting many of these cases to this fortunate termination, I had the aid of several intelligent officers, who were attached to me, and from whom I derived much assistance. Some of them are now stationed in countries where ophthalmia exists in its severest forms, and I hope shortly to hear from them a favourable account of their success in its treatment, especially in the acute stage, by the solution of caustic and moderate depletion.

In taking the preceding review of the ocular diseases and operations that have occurred at this establishment for the last two years, I have endeavoured to render it as brief as possible, and aimed more at compressing the matter, than in giving an elaborate and finished detail; as I know too well the important occupations of those who will have to peruse it, to trespass longer on their valuable time than is absolutely necessary. If, in attempting to accomplish this desirable end, I may appear in some parts obscure, I trust it will be viewed with indulgence; and, should any explanation be required on any of the points of practice, I shall be most happy to afford it.

*Yearly Report of Ocular Diseases, from 21st December, 1822, to 20th December, 1823.*

| *Numerical Abstract of principal Diseases.* | | *Of these, Re-admissions.* | *Number of Men treated.* |
|---|---|---|---|
| Remained, 20th December, 1822 | 46 | 0 | 46 |
| Since admitted | 138 | 18 | 120 |
| Total treated | 184 | 18 | 166 |
| Discharged: To join regiments | 71 | 2 | 69 |
| Discharged: Unfit from eye diseases | 65 | 15 | 50 |
| Discharged: Unfit from other causes | 24 | 1 | 23 |
| Total | 160 | 18 | 142 |
| Remaining | 24 | 0 | 24 |

*Return of Operations performed, together with their Results.*

| | | |
|---|---|---|
| Artificial Pupil | Successful | 6 |
| Do. with Cataract | Successful | 1 |
| | Unsuccessful | 1 |
| Cataract | Successful | 4 |
| | Unsuccessful | 2 |
| Tumor of Lids | Successful | 1 |
| Staphyloma | Successful | 1 |
| | Total | 16 |

The two unsuccessful cases of cataract succeeded perfectly as to the operation, but no benefit ensued, the eyes being amaurotic; which was strongly apprehended to be the case, previous to operating.

